# Supplementary material for: Impaired voice processing in reward and salience circuits predicts social communication in children with autism
Source: eLife. 2019 Feb 26;8:e39906. doi: 10.7554/eLife.39906 (PMC6391069; doi:10.7554/eLife.39906)
Supplement: Transparent reporting form [file elife-39906-transrepform.docx]

***eLife’s* transparent reporting form**

We encourage authors to provide detailed information *within their submission* to facilitate the interpretation and replication of experiments. Authors can upload supporting documentation to indicate the use of appropriate reporting guidelines for health-related research (see [EQUATOR Network](http://www.equator-network.org/%20)), life science research (see the [BioSharing Information Resource](https://biosharing.org/)), or the [ARRIVE guidelines](http://www.plosbiology.org/article/info:doi/10.1371/journal.pbio.1000412) for reporting work involving animal research. Where applicable, authors should refer to any relevant reporting standards documents in this form.

If you have any questions, please consult our Journal Policies and/or contact us: [editorial@elifesciences.org](mailto:editorial@elifesciences.org).

**Sample-size estimation**

- You should state whether an appropriate sample size was computed when the study was being designed
- You should state the statistical method of sample size computation and any required assumptions
- If no explicit power analysis was used, you should describe how you decided what sample (replicate) size (number) to use

Please outline where this information can be found within the submission (e.g., sections or figure legends), or explain why this information doesn’t apply to your submission:

Our sample size (42 participants; 21 participants in each group) was chosen based on the following factors:

(1) Best-practice reviews have suggested that groups in neuroimaging studies should have > 20 participants (Button et al., 2013).

(2) Our previously published task-based imaging studies of children with ASD have been sufficiently powered using sample sizes that are comparable to those used here (e.g., Iuculano et al., *Biol Psychiatry*, 2013 (*N*=36; *N*=18 in each group); Uddin et al., *Cereb Cx*, 2014 (*N*=34; *N*=17 in each group); Odriozola et al., *Soc Cogn Affect Neurosci*, 2016 (*N*=40; *N*=20 in each group)).

(3) Recent high-impact imaging studies from other research groups that have investigated social stimulus processing in individuals with ASD have used sample sizes that are comparable to those used here (Gordon et al., *Sci Reports*, 2016 (*N*=20 in full ASD group); Andari et al., *Cortex*, 2016 (*N*=20 in ASD group); Tam et al., *Sci Reports*, 2017 (*N*=22 in ASD group; *N*=24 in TD group); Rosenblau et al., *Soc Cogn Affect Neurosci*, 2016 (*N*=20 in ASD group; *N*=21 in TD group)).

**Replicates**

- You should report how often each experiment was performed
- You should include a definition of biological versus technical replication
- The data obtained should be provided and sufficient information should be provided to indicate the number of independent biological and/or technical replicates
- If you encountered any outliers, you should describe how these were handled
- Criteria for exclusion/inclusion of data should be clearly stated
- High-throughput sequence data should be uploaded before submission, with a private link for reviewers provided (these are available from both GEO and ArrayExpress)

Please outline where this information can be found within the submission (e.g., sections or figure legends), or explain why this information doesn’t apply to your submission:

We have included all details of the experiments, inclusion criteria, and identification of outliers in the “Participants,” “fMRI Task,” and “Movement criteria for inclusion in fMRI analysis” subsections of the Methods.

**Statistical reporting**

- Statistical analysis methods should be described and justified
- Raw data should be presented in figures whenever informative to do so (typically when N per group is less than 10)
- For each experiment, you should identify the statistical tests used, exact values of N, definitions of center, methods of multiple test correction, and dispersion and precision measures (e.g., mean, median, SD, SEM, confidence intervals; and, for the major substantive results, a measure of effect size (e.g., Pearson's r, Cohen's d)
- Report exact p-values wherever possible alongside the summary statistics and 95% confidence intervals. These should be reported for all key questions and not only when the p-value is less than 0.05.

Please outline where this information can be found within the submission (e.g., sections or figure legends), or explain why this information doesn’t apply to your submission:

All details regarding the statistical analysis, as specified above, can be found in the “Voxel-wise analysis of fMRI activation,” “Brain-Behavior Analysis,” “Brain Activity Levels and Prediction of Social Function,” and “Effective Connectivity, Group Classification, and Prediction of Social Function” subsections of the Methods.

(For large datasets, or papers with a very large number of statistical tests, you may upload a single table file with tests, Ns, etc., with reference to sections in the manuscript.)

**Group allocation**

- Indicate how samples were allocated into experimental groups (in the case of clinical studies, please specify allocation to treatment method); if randomization was used, please also state if restricted randomization was applied
- Indicate if masking was used during group allocation, data collection and/or data analysis

Please outline where this information can be found within the submission (e.g., sections or figure legends), or explain why this information doesn’t apply to your submission:

All details regarding how samples were allocated into experimental groups can be found in the “Participants” section of the Methods.

**Additional data files (“source data”)**

- We encourage you to upload relevant additional data files, such as numerical data that are represented as a graph in a figure, or as a summary table
- Where provided, these should be in the most useful format, and they can be uploaded as “Source data” files linked to a main figure or table
- Include model definition files including the full list of parameters used
- Include code used for data analysis (e.g., R, MatLab)
- Avoid stating that data files are “available upon request”

Please indicate the figures or tables for which source data files have been provided:

All fMRI activation maps reported in the manuscript will be made available at neurovault. Full single subject raw data will be made public on the NIH NDAR repository, as per NIH rules (procedure is ongoing).
